# Higher Toughness of Metal-nanoparticle-implanted Sodalime Silicate Glass with Increased Ductility

**DOI:** 10.1038/s41598-019-51733-5

**Published:** 2019-10-28

**Authors:** Madoka Ono, Satoshi Miyasaka, Yoichi Takato, Shingo Urata, Haruhiko Yoshino, Ryota Ando, Yasuo Hayashi

**Affiliations:** 1 AGC Inc., Materials Integration Laboratories, Yokohama, 221-8755 Japan; 2 Hokkaido University, Research Institute for Electronic Science Laboratory of Nanostructured Functional Materials, Kita 20 Nishi 10, Kita-ku, Sapporo 001-0020, Hokkaido, Japan; 3 AGC Inc., Innovative Technology Laboratories, Yokohama, 221-8755 Japan

**Keywords:** Glasses, Mechanical engineering

## Abstract

In this report, we propose a novel framework for toughening brittle oxide glass originated from enhanced ductility by implanting a secondary material comprising different mechanical properties. To do so, copper-metal nanoparticles are implanted into the subsurface layer of commercial soda-lime silica glass by using the electrofloat method. The crack initiation load of the implanted glass is found to be comparable to the glass chemically strengthened in ordinary tempering conditions. By observing crack propagation and stress distribution from cross-section, it is found that the crack propagation stops within the metal nanoparticle implanted layer, due to the stress dissipation or relaxation. The copper-implanted glass shows improved toughness with decreased hardness. The toughening mechanism of the composite glass is theoretically studied using molecular dynamics calculations on an amorphous silica model with copper nanoparticles embedded, and Peridynamics fracture simulations for indentation on a glass sheet model whose surface was implicitly modeled as the copper-implanted oxide glass. The experimentally observed phenomena of intrinsic toughening were well explained by the series of the conducted simulations.

## Introduction

Glass is usually highly homogeneous. Due to the homogeneity, glass can freely be formed into arbitrary shapes, or easily be mass-produced in uniform conditions. Such availability is industrially favorable, and hence glass has been one of the affordable, prevalent industrial materials. But because of the homogeneity, and there are countless number of microcracks existent on its surface, a glass experiences large tensile stress at the crack tip when its surface is subjected to a tensile load. A pristine glass fractures very easily not only due to their original brittleness as a material, but as the result of its homogeneity^1^.

To avoid initiation and extension of a crack at the crack tip in a pristine glass, one can compensate tensile stress at the surface of the glass by tempering glass physically^2^ or chemically^3,4^. These treatments induce compressive stress in the vicinity of glass surface. It reduces the tensile stress that initiates crack propagation, so that stress accumulation at the tip is reduced. On the other hand, avoiding such stress concentration at the crack tip is believed to be another way to strengthen the glass. For example, creating heterogeneity by introducing core-shell structures inside metallic glass is adopted as advantageous to diffuse stress and increase toughness. Such toughening process is called as “intrinsic toughening”^5^. However, with respect to oxide glasses, the approach of making heterogeneity had been limited to introducing crystalline-phase microstructures^6–10^ or phase-separation where the heterogeneity enhances fracture toughness through increasing fracture surface roughness so that the propagation energy is consumed by making a new surface^11,12^. These methods are practically available, however it is difficult to control the amount of the secondary phases because both crystallization and phase separation utilize transition of thermal equilibrium condition. Furthermore, the amount and composition of the secondary phases depend on the parent glass, so that overall properties of the composite materials are somewhat uncontrollable.

On the contrary, additives, such as metals and ceramics, are possible ingredients to modify properties of the oxide glass without vitiating feature of parent glass. Especially, a variety of methods are known to induce partial metalization in oxide glass such as those used in stained glasses^13–17^. As one of those methods, we apply the electrofloat method to implant nano-scale metal particles into the subsurface of the oxide glass superimposed by direct-current voltage. Our study shows, for the first time, that implanting metal nanoparticles can be one of the methods to “intrinsically toughen” soda-lime silica glass (SLG). The metal nanoparticles implanted glass shows crack initiation load (CIL) so large as to be comparable to the chemically strengthened one by the ion-exchange (IOX) method in normal condition. The obtained glass composite shows characteristic features, such as lower Vicker’s hardness. We obtain a clear evidence that stress is released in the metal-particle layer by observing the stress distribution from the cross-section. Besides the experimental investigations, both atomistic and continuum simulations are conducted to understand advantages of the mechanical property of glass modified with metal nanoparticles. A combination of reactive molecular dynamics (MD) simulation for the microscale and Peridynamics for the macroscale is demonstrated to be effective for reproducing and explaining the experimentally observed phenomena.

## Results and Discussions

### Configuration of metal-implanted SLG made by the electrofloat method

**Figure 1:**
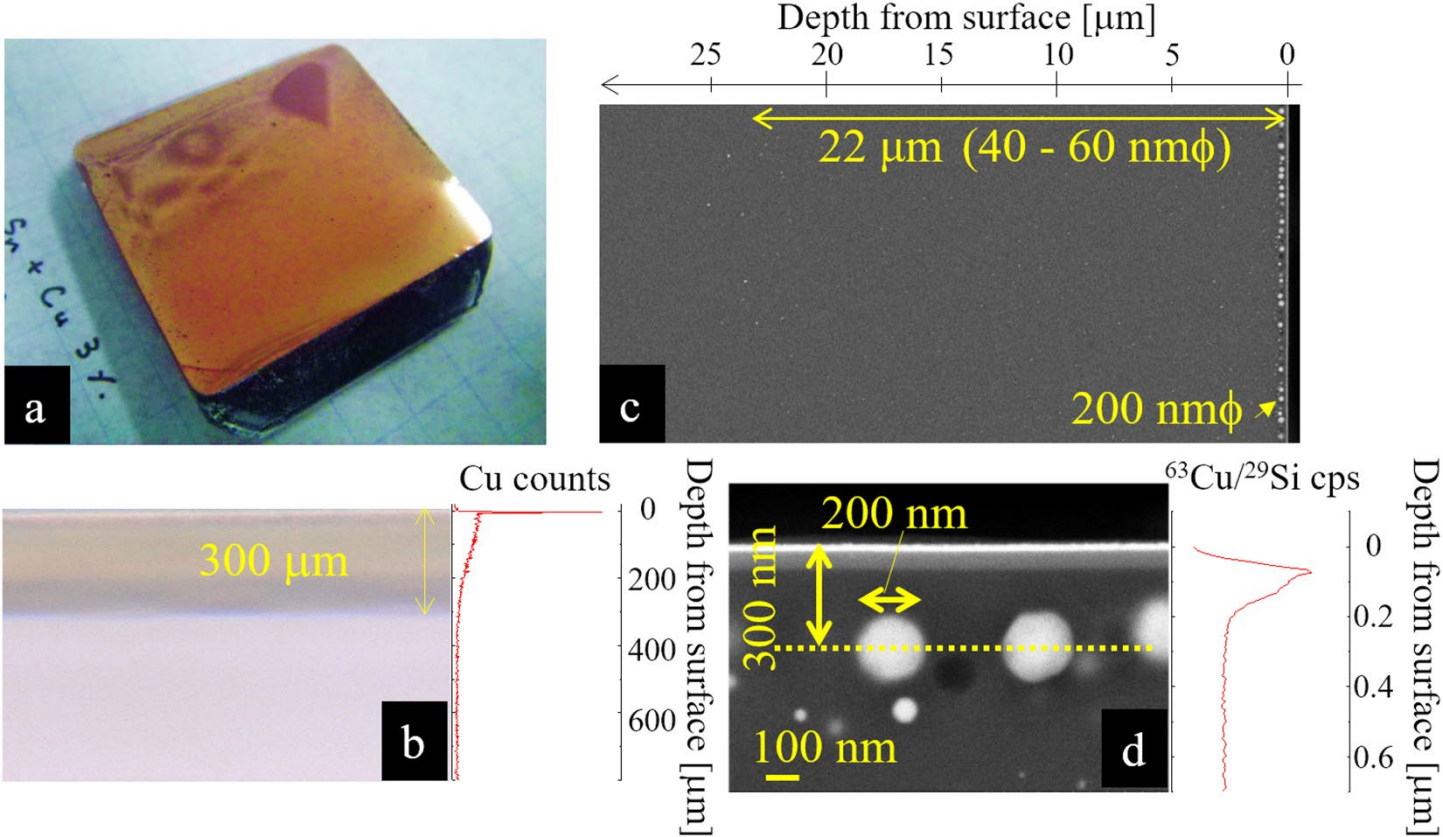
(**a**) Copper-implanted soda-lime silica glass prepared by electro-float method. (**b**) A cross-sectional view taken by optical microscope. The profile on the right shows Cu intensity profile measured by EPMA. (**c**) Scanning electron microscope image of the cross-section from the surface of the sample to the depth of about 30 μm. (**d**) Scanning electron microscope image of the cross-section near the surface. The profile on the right shows Cu intensity profile measured by SIMS. Here, the thin white layer at the surface seen on (**c**) and (**d**) are due to Pt evaporated onto the sample surface in order to do the SEM-observation.

The appearance of the SLG sample made by the electrofloat method is shown in Fig. 1(a). The surface of the sample had high reflectance with brownish color. From the cross-sectional view by optical microscope, as shown in Fig. 1(b), the brownish copper layer was observed in the  SLG surface down to approximately 300 μm. The profile on the right of Fig. 1(b) shows Cu intensity measured by electron probe micro analyzer (EPMA). There was a high intensity layer of Cu close to the surface while gradual decrease due to ion diffusion was seen in the deeper layer. From the color of cross-sectional transmission picture by microscope, the highly refractive brownish color on the surface layer is supposed to be due the subsurface layer with high copper intensity.

To observe the layer with high copper intensity, scanning electron microscope (SEM) image of the cross-section near the surface was taken as in Fig. 1(c). A monolayer of larger copper particles exist close to the surface while smaller particles exist sparsely in the deeper area. Smaller metal nanoparticles were observed down to approximately 20 *μ*m in depth as seen in Fig. 1(c). Figure 1(d) magnifies Fig. 1(c). It was shown that the diameter of the larger particles are approximately 200 nm, while smaller nanoparticles have diameter of about 50 nm. The center of the larger copper layer is located at about 300 nm in depth from the surface. On the right side of Fig. 1(d), copper intensity profile measured using secondary ion mass spectrometry (SIMS) is shown. From the copper profile, it was also implied that copper density is high at the monolayer but abruptly decreases within 1 *μ*m region, and then gradually decreases by going inward within 300 *μ*m. It has been known that, by the electrofloat method, Na^+^ moves into inner surface of the SLG sample and, in turn, Cu^+^ intrudes into glass from Cu:Sn melt. Density profiles of the other ions, such as tin, calcium, magnesium, sodium and etc. are presented in supporting information (Fig. S1). We suppose the intruded Cu^+^ transforms to Cu metal by the reducing reaction of Sn Sn_2_^+^ + 2Cu^+^ → Sn_4_^+^ + 2Cu^0^. From the color and the profile in Fig. 1(b,c), Cu metal particles are supposed to exist down to 20 *μ*m, while copper ions exist down to 300 *μ*m.

The metal nanoparticle implanted in SLG made by the electro-float method is known to have oxidized interface as CuO or CuO_2_. By using reactive molecular dynamics simulation, such metal nanoparticles have strong adhesive interaction with glass matrix by chemical bonding generated between Cu-O and Si^18^. Thus, adhesion between metals and oxide glasses should be strong in the Cu-implanted SLG studied in this work.

### Effect of copper nanoparticle in SLG on mechanical strength

**Figure 2:**
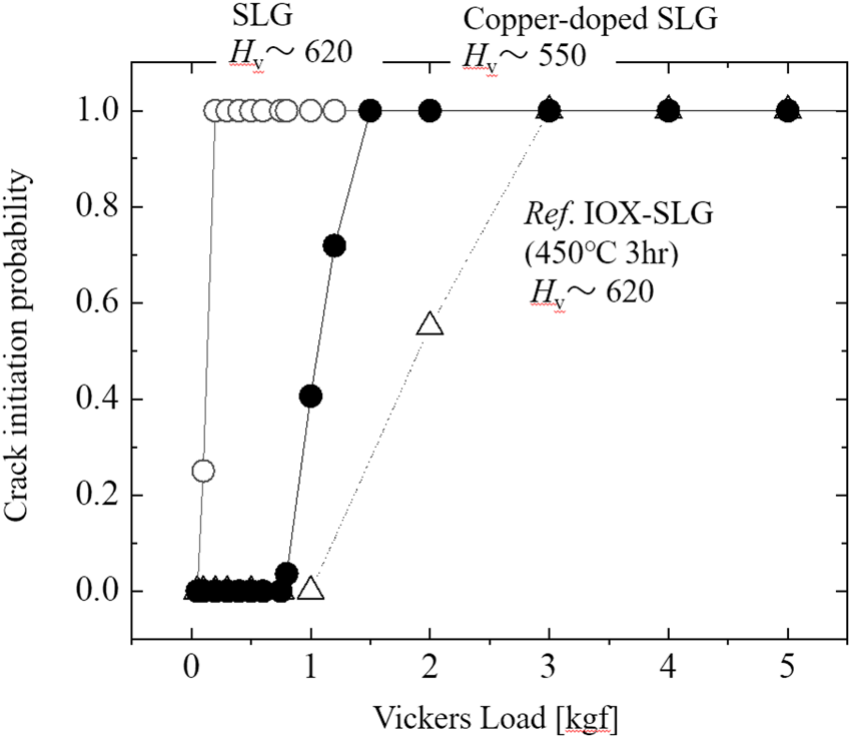
Crack initiation probability against the load (CIL) of (○) reference soda-lime silica glass, (•) copper-nanoparticle-implanted soda-lime silica glass and (Δ) ion-exchanged soda-lime silica glass, which was ion-exchanged at 723 K for 3 hours. Pictures of the example of footprints of the vickers indentation on Cu-doped SLG and reference SLG are shown in Fig. S3.

Figure 2 shows the crack initiation probability against the loading Vickers indenter, called CIL. The tested samples include nanoparticle implanted SLG (filled circle), SLG with the same heating process but no application of voltage so that no nanoparticle is implanted (open circle). Usually, SLG shows very low CIL exerted by the behavior, such that cracks appear by indenting 0.1 kgf. On the contrary, no crack was observed even under 0.8 kgf in the copper-doped SLG. The CIL behavior in the copper-doped SLG was even close to ion-exchanged SLG, which is shown by triangles in Fig. 2. The process condition for ion-exchange is 723 K for 3 hours in 100% KNO_3_, which is common for SLG. Therefore, it indicates that copper nanoparticle doping has a strengthening effect comparable to ion-exchange. To examine the reason for the high CIL, Vicker’s hardness, *H*_v_ was measured by observing the size of the indentation footprint at the amount of load of 0.1 kgf^19^. Usually *H*_v_ of SLG is around 620–650. As the matter of fact, *H*_v_ of the prepared SLG for reference was 620. The value is relatively small for SLG due to its rapid quenching at high temperature as to mimic the heat treatment condition of Cu-implanted one. Such quenching makes the surface of the SLG sparser and softer. Cu–implanted SLG showed distinctively smaller *H*_v_ of 550 even when compared to the quenched SLG. It is worth noting that chemically tempered SLG (IOX-SLG) tends to show higher *H*_v_ especially at surface layer where potassium exchanges sodium (See Fig. S2 in SI). That implies that harder surface is not always necessary to make glass stronger. Softer surface can be so strong as that with harder surface.

**Figure 3:**
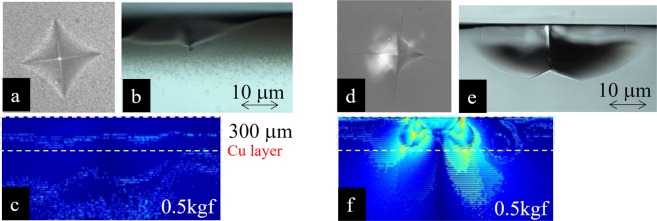
(**a**) A footprint of the Vickers indentation for the Cu–implanted SLG with indentation load of 0.5 kgf. (**b**) A cross-sectional observation of indentation footprint after indentation in Cu-implanted SLG. A crack was observed at the bottom of the indentation, but the propagation stopped in the Cu-implanted layer. (**c**) *In-situ* stress distribution observed using birefringence method from cross-sectional view in the Cu-implanted SLG. (**d**) A footprint of the Vickers indentation for the reference SLG with indentation load of 0.5 kgf. (**e**) A cross-sectional observation of indentation footprint after indentation in the reference SLG. Median cracks are observed to extend up to the surface. (**f**) *In-situ* stress distribution observed using birefringence method from cross-sectional view in the reference SLG. The color range of the stress is set to be the same as (**c**).

The effect of Cu-implantation in fracture toughness, *K*_c_ was also evaluated by measuring the length of cracks, which extend from four corners of Vicker’s indentation footprint, and compared with that of the reference SLG. The detail of this measurement method of *K*_c_ is described in refs^20,21^. The *K*_c_ value for Cu–implanted SLG was *K*_c_ = 1.5 MPa·m^1/2^, which was obviously larger than the normal SLG whose *K*_c_ was 0.8 MPa·m^1/2^ ^22^. In Fig. 3(a,d), we show the example of the footprints of the Vickers indentation for the Cu-implanted SLG and the reference SLG with indentation load of 0.5 kgf, respectively. The Cu-implanted SLG shows larger indentation footprint than that of the reference SLG, which indicates the softer surface, and no crack is seen at the corner of the indentation. On the contrary, the reference SLG showed long cracks at all corners of the indentation footprint. We observed how a crack initiates and propagates inside the samples by *ex-situ* cross-sectional observation using optical microscope. As seen in Fig. 3(b), the crack stopped propagating in the layer where Cu nanoparticles are implanted. Cu nanoparticle-implanted glass also showed median crack generation and propagation with higher load. However, as explained in SI Fig. S4, the crack tends to stop propagating when it enters the metal implanted layer. It is obviously different from the reference SLG, where the median crack is generated from the bottom of the indentation and propagated through glass up to the surface (Fig. 3(e)).

To clarify the mechanism of preventing crack propagation, *in-situ* stress distribution of these two samples were compared using birefringence method from cross-sectional view. Figure 3(c,f) are images taken while the indenter is inserted at the similar timing. The absolute value of stress is not evaluated because the birefringence images correspond to the summation of stress across the sample. But as the thicknesses of the two samples were the same, Fig. 3(c,f) can be directly compared. It is obviously indicative that the stress visible under the Cu-implanted layer is low. (Note that the birefringence may not be well observable as the reference SLG due to the color of the implanted metal-particles). On the contrary, the reference SLG showed much larger stress distribution under the indenter down to deeper area. Therefore, the suppression of the stress under Vickers indentation in Cu-implanted SLG is supposed to be the reason for the higher CIL in the Cu-implanted SLG. This strength must be partly supported by strong adhesive interaction between Cu nanoparticles with the glass matrix. Lower *H*_v_ should contribute to the suppression of the stress distribution. However, considering that the Young’s modulus of crystalline Cu is higher than SLG, it is not obvious that the Cu-nanoparticle implanted SLG has lower modulus.

To verify the hypothesis derived from the experiments, we examine the effect of Cu-particle implantation in glass on the crack resistance using Peridynamics simulations informed by molecular dynamics simulationson Cu-particle embedded models.

### Mechanical properties of the copper nanoparticles implanted glass via MD simulation

**Figure 4:**
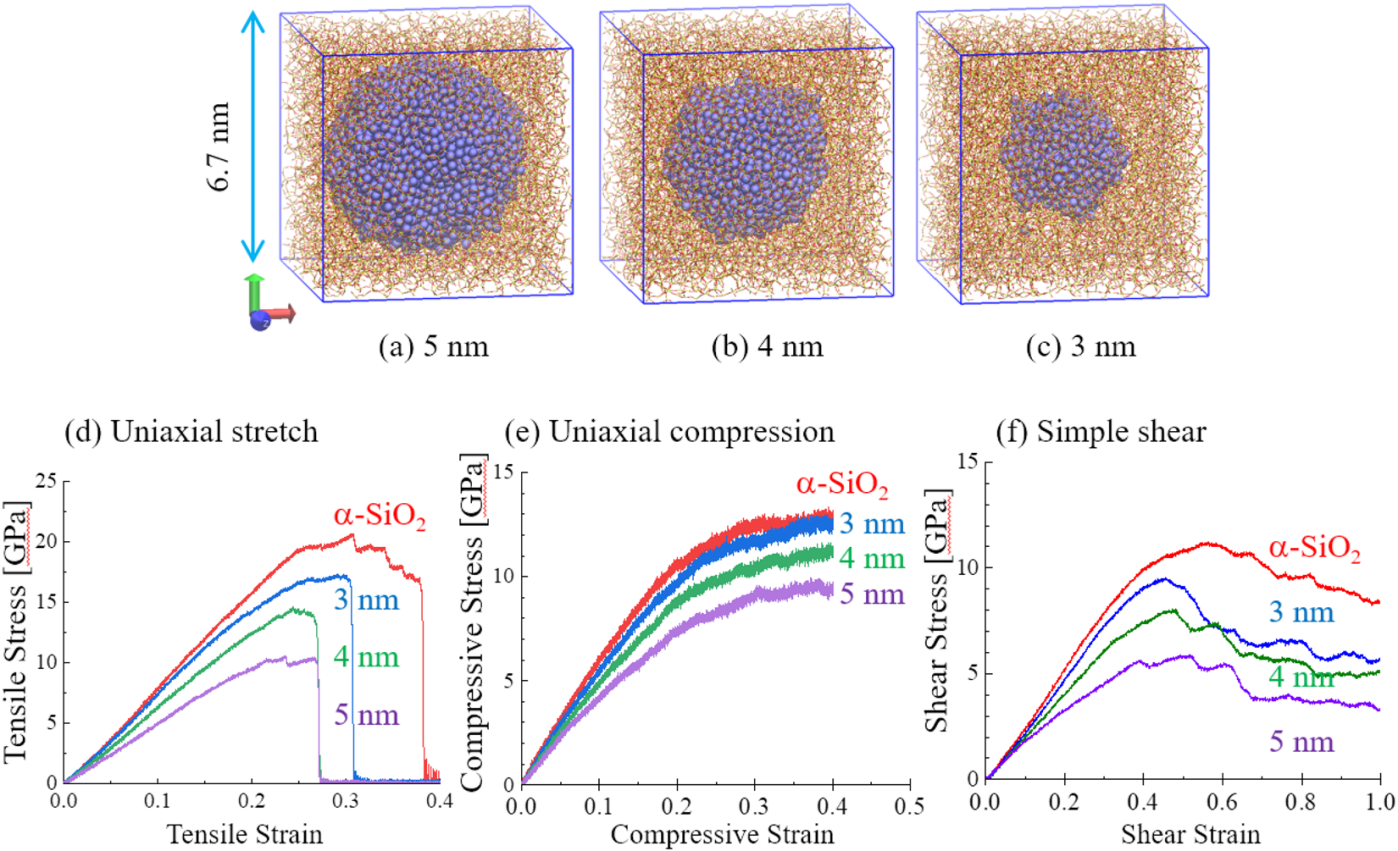
Atomistic models for amorphous silica with inclusion of a copper-nanoparticle. Line draws network of Si-O and blue sphere is copper atom. (**a**–**c**) show models for amorphous silica glass with copper particles whose diameters are 5, 4 and 3 nm, respectively. (**d**) through (**f**) show stress-strain curves for (**d**) uniaxial strech, (**e**) compression and (**f**) shear deformation for the composite models. The simulation result for amorphous silica (*α*-SiO_2_) is also drawn for comparison.

**Figure 5:**
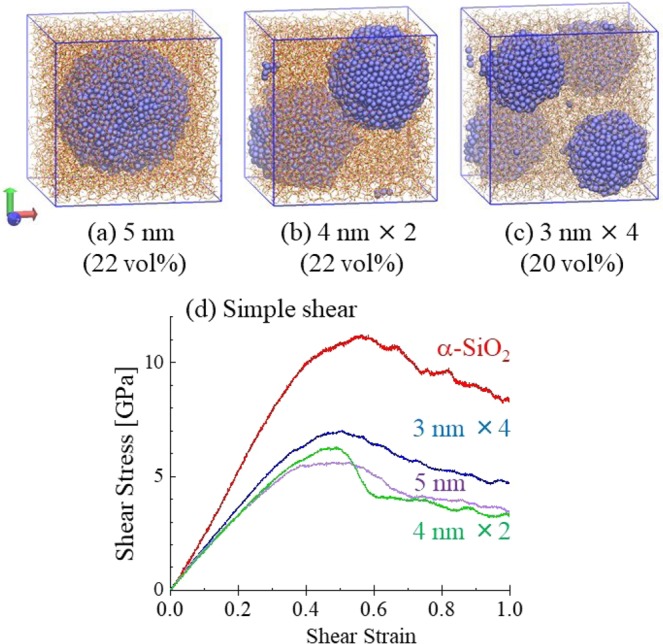
Atomistic models for amorphous silica with inclusion of copper-nanoparticles, whose total volume ratios are similar. Lines draw the  network of Si-O and blue sphere are copper atoms. (**a**–**c**) show models for amorphous silica glass with copper particles whose diameters and numbers are 5 nm and 1, 4 nm and 2, and 3 nm and 4, respectively. (**d**) shows stress-strain curves for shear deformation calculated for the composite models. The simulation result for amorphous silica (*α*-SiO_2_) is also drawn for comparison.

To understand the mechanical properties of the copper nanoparticles implanted glass, we performed deformation in simulations on the composite models consist of a copper nanoparticle and amorphous SiO_2_ (*α*-SiO_2_) as shown Figs 4 and 5. The model was made by the ReaxFF model^23,24^. The models including a nano-copper particle with diameter of 3, 4 and 5 nm were compared to examine volume effect of secondary copper phase (Fig. 4(a–c)). Their volume ratios of copper are approximately 5, 11 and 22%, respectively. In addition, two models, whose total copper volume ratios are approximately the same with the 5 nm copper-particle model, were investigated to clarify the particle size effect on the mechanical properties. One includes two particles with diameter of 4 nm (Fig. 5(b)) and the other one includes four particles with diameter of 3 nm (Fig. 5(c)).

We measured stress-strain curves for uniaxial stretch, compression and simple shear deformations and investigated the nanoparticle effect on the mechanical responses. For the uniaxial and the shear deformation simulations, 1 × 10^−6^ ps^−1^ and 1 × 10^−5^ ps^−1^ of strain rates were assumed, respectively. The stress-strain curves for the deformations in comparison with *α*-SiO_2_ models are summarized in Fig. 4(d–f). In any cases, stresses of the nanoparticle inclusion models are lower than that of the *α*-SiO_2_ model. The moduli monotonically decline with increasing nanoparticle volume ratio, and the rate of diminution is larger for higher copper volume ratio as summarized in Table 1. Indeed, the 5 nm size of copper particle declines the moduli and maximum/minimum stress in about 30–40% and 25–50%, respectively, even though the volume ratio is 22%. It is thus inferable that more metal precipitation is more effective to modify the mechanical properties of oxide glass.

**Table 1:** Moduli and maximum or minimum stresses of the copper-implanted models evaluated using molecular dynamics simulations, which are shown in GPa unit. Percentages are ratios of the properties to those of amorphous silica (α-SiO_2_).

Cu volume ratio [vol%]	*α*-SiO_2_	3 nm	4 nm	5 nm	3 nm × 4	4 nm × 2
	0	5	11	22	20	22
	Modulus [GPa]					
Stretch	70.4	66.1	57.2	47.5	48.4	47.3
		93.9%	81.3%	67.6%	68.8%	67.2%
Compress	65.3	62.3	53.3	47.5	50.0	50.1
		95.5%	81.7%	72.8%	76.6%	76.7%
Shear	26.3	24.2	20.5	16.4	17.8	16.5
		91.9%	78.2%	62.3%	67.6%	62.9%
	Maximum or minimum stress [GPa]					
Stretch	20.7	17.3	14.6	10.6	12.4	10.9
		83.8%	70.5%	51.3%	59.9%	52.7%
Compress	−13.4	−13.0	−11.7	−10.0	−9.5	−9.7
		97.1%	87.2%	74.5%	71.4%	72.9%
Shear	11.2	9.5	8.1	5.9	7.1	6.2
		84.9%	71.9%	52.4%	63.3%	55.4%

According to these results, it is presumed that the Young’s modulus and shear modulus are decreased by implanting Cu nanoparticles even though the moduli of Cu itself are higher than those of glass. It is expected that amorphousization of copper metal softened the composite effectively. Furthermore, it seems that the nanoparticle is more influential to the shear modulus than to the Young’s modulus, implying that the secondary material of metal particle promotes shear deformation and implements plasticity to the brittle oxide glass matrix.

Three models possessing approximately the same copper volume ratio are compared in Fig. 5. One of the models containes a 5-nm particle, while the other contains two 4-nm particles, and the last contains four 3-nm particles. It was found that the larger particle decreases shear modulus more effectively (Fig. 5(d)). Even though our atomistic simulations limit the particle size to a few nanometers, it infers such nanoparticle effect should be apparent in the case of particles with diameter of several hundreds nanometers, as experimentally observed in Fig. 1(d).

To confirm the emergence of dislocation in the deformed nanoparticle models, we measured local shear deformations at each copper atom when shear strain is 0.25 as shown in Fig. S5 in the supporting information. The algorithm of the analysis used here can be found in ref.^25^. According to the figure, one can find that the models with 4 and 5 nm in diameter clearly demonstrate a shear band running in the middle of the copper nanoparticle, indicating that even the models with nanometer-scale particles are large enough to have dislocations in the metal-particle implanted glass. Thereby, we infer that the larger metal particles experimentally observed in this study should have a more pronounced effect that softens the hybrid glass.

Overall, due to the higher plasticity of amorphous Cu, Cu nanoparticles together with the glass matrix start to deform plastically when the force is applied above yield strength of Cu, which ends up as a substantially heterogeneous material with lower moduli and higher plasticity. We will study the effect of the enhanced plasticity of the surface layer on the crack resistance using macroscopic simulations in the following section.

### Crack initiation via Peridynamics simulation

To verify the conjecture for the toughening mechanism inferred by our MD simulations for the nanoparticle inclusion model, we carried out indentation simulations for a simplified macroscopic composite model by means of Peridynamics based on a non-local continuum theory. According to the aforementioned MD simulations and Vickers indentation experiments, the nanoparticle precipitated model is in essence softer than its parent SLG as stated in Table 1. We speculate that the softening arising from the lower Young’s modulus may hinder crack initiation and propagation during indentation. Here we do not take into account plasticity since the substantial mechanical responses to the external loads obtained by the earlier MD simulations are elastic if strain is not too high.

**Figure 6:**
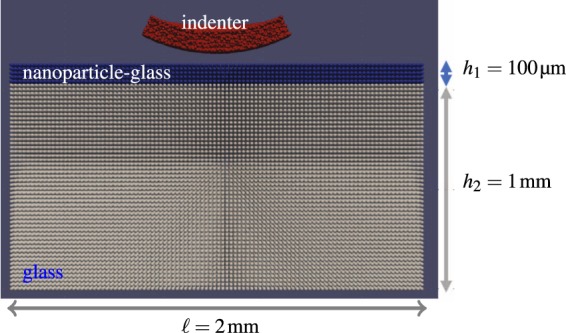
Macroscopic indentation model for the composite glass used for Peridynamics simulation. A rigid, spherical indenter of radius *R* = 0.8 mm is moved at a constant displacement toward the top surface of the rectangular block. Its dimensions are a length of $\ell =2\,{\rm{mm}}$, a width of *w* = 2 mm, and a height of *h* = 1.1 mm.

Based on this hypothesis, we built a Peridyanamics model consisted of a soft thin layer on top of a slab of stiff glass as depicted in Fig. 6. The soft layer represents the nanoparticle-implanted glass, and its thickness is set at 100 μm, which is of the order of the value estimated from our experimental measurement (see Fig. 1(b,c)). The composite model is indented by a spherical indenter, which is the so-called Hertzian indentation experiment. Unlike the Vickers indenters, this blunt indenter does not induce plastic deformation, due to a relatively large contact surface area formed between the spherical indenter and the indented material and resulting low contact pressure. The setup allows to purely probe the effect of the variation of Young’s modulus on cracking since plasticity is intentionally excluded.

Young’s moduli for SLG and the composite layers are set at 70.4 GPa and 47.5 GPa (67.6%), respectively, as the MD simulations suggested. Poison ratio is assumed to be 0.25. To enable crack initiation and propagation, the critical energy release rates for the two materials, *G*_*c*_ = 8.5 J/m^2^, are used on the basis of the fracture toughness, *K*_IC_ = 0.8 MPa·m^1/2^, of a typical glass and the relation *G*_*c*_ = *K*_IC_^2^/[*E*/(1 − *ν*^2^)]. The model is indented with a spherical rigid indenter of radius 0.8 mm moving at a constant displacement of 5 m/s.

**Figure 7:**
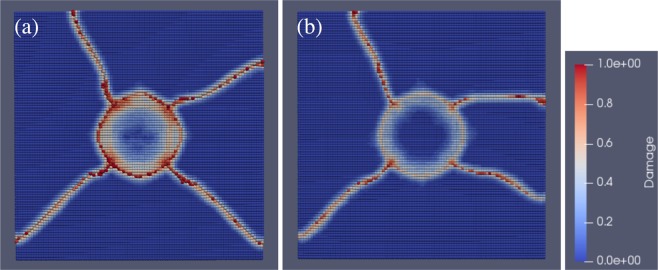
Damage of the materials considered after indentation computed by Peridynamics. Soda-lime silica glass is shown in (**a**) and the nanoparticle-implanted glass in (**b**) for the top surface. The colorbar describes the material damage ranging from zero (blue) to one (red), and the damage equal to 0.4 amounts to crack formation. For a better representation of damage, the snapshots were taken when cracks on each surface are well developed.

Figure 7 shows a comparison of material damage caused by indentation between the SLG and the composite. Note that the small spheres represent macroscopic material points produced as a result of discretization of the domains for Peridynamics computation, and therefore they are not atoms unlike the ones seen in the prior MD section. The damage level is described by a value that ranges between zero (blue) and one (red). These snapshots are taken when damage of any material points reaches unity. The glass block in Fig. 7(a) has a ring crack colored in red that appears on the top surface where the maximum tensile stress builds up^26^ and has radial cracks as well extended from the circumference of the ring crack. In contrast, the two-layer model that represents the composite in Fig. 7(b) evidently has less damaged areas.

**Figure 8:**
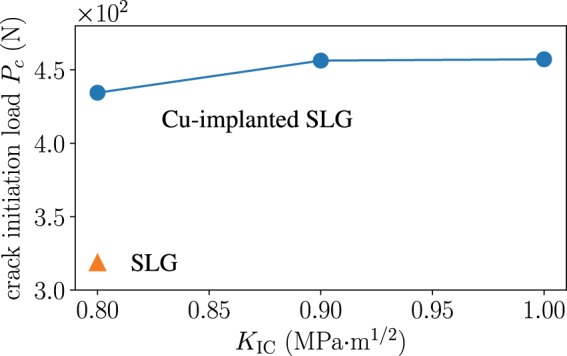
Effect of the implantation of copper nanoparticles in the vicinity of the glass surface examined by Peridynamics simulations. The critical initation load, *P*_*c*_, is plotted as a function of the fracture toughness, *K*_IC_. Our Peridynamics simulations show that the crack initiation load increases solely by softening the glass surface even if the fracture toughness remains unchanged. The increase of the fracture toughness improves the crack initiation load, as expected.

The difference in damage between the SLG and the composite layers implies that the presence of the soft layer alleviates the tensile stress accumulation that causes ring crack initiation. The results of these macroscopic simulations are consistent with the stress delocalization revealed by our experiments. We further evaluated the crack initiation load, *P*_c_, by observing the local behaviors seen in Peridynamics simulations. Here the crack initiation load is obtained when a ring crack starts to form on the surface. The criterion for the crack formation is based on a local damage value of 0.4 ^27^. As shown in Fig. 8, the calculated *P*_c_ of the composite model is 4.3 × 10^2^ N. This value is evidently higher than the value of SLG, 3.1 × 10^2^ N. Therefore, the softening alone gives rise to the higher crack resistance. The macroscopic simulation results qualitatively agree with the experiments.

The increase in *P*_c_ is a lower bound since we made an assumption that the fracture toughness stays the same regardless of nanoparticle additives. But the actual fracture toughness *K*_IC_ of the copper-implanted glass is presumably higher according to the experimental values of *K*_c_ for those glasses stated earlier in this paper. We then carried out additional simulations for higher, reasonable values of *K*_IC_ = 0.9, 1.0 MPa·m^1/2^. Figure 8 indicates that the softening arising from the lower Young’s modulus is a dominant factor in the parameter ranges considered though *P*_*c*_ increases slightly for the materials having higher *K*_IC_.

## Conclusions

We show, for the first time we believe, that toughening of SLG by making it heterogeneous with copper-nanoparticle implantation is possible. By using electrofloat method, SLG with copper-nanoparticles of diameter of about 200 nm were implanted close to the surface while particles of 40 to 60 nm *ϕ* were implanted sparsely down to about 20 *μ*m in depth from the surface was obtained. Crack initiation load of the obtained Cu-nanoparticle-implanted SLG apparently improved to be even comparable to that of normally ion-exchanged SLG. The obtained composite SLG showed improved fracture toughness with lower Vickers hardness. From *ex-situ* and *in-situ* cross-sectional measurement, we confirmed that the crack stops inbetween the Cu-implanted layer where stress was observed to be obviously suppressed. In order to investigate the toughening mechanism of the composite glass, theoretical approach using MD and Peridynamics simulations were conducted. It was indicated that, even though the implanted metal originally had larger modulus than the mother glass, the modulus, especially shear modulus of the composite glass can be lower. By Peridynamics, it was also implied that composite glass with thin layer of such lower modulus can induce delocalization of stress under indenter which finally increase the crack initiation load, more effectively than the increased fracture toughness. The simulation of MD has several additional indications; as larger Cu-particle was more effective to suppress stress intensity and introduce plastic deformation, the size of the metal should not be too small in order to induce the toughening effect. Also, it is indicative that not all the copper particles with depth of 300 *μ*m, which unfortunately makes the glass opaque, is necessary to keep the strength.

The point of the toughening mechanism is the softening of the SLG by the metal nanoparticle implantation which induces stress dissipation. Because the modulus of metal is larger than that of glass matrix, the resultant SLG with lower modulus with larger deformation should be attributed to ductility due to metal particles which takes place when the load exceeds the yield strength. This toughening mechanism is distinguishable to the other hybrid glass system such as glass with phase-separation, and glass ceramics^28,29^ because they increase fracture surface to impede total breakage. It is also largely different in scale from glass with nanoductility^30^ induced by disconnecting bonds of network atoms either by introducing alkali ions or disconnecting bonds by electron beam irradiation.

Considering that the electrofloat method is industrially applicable, to not only SLG but also to various kinds of glass, we believe that the metal-nanoparticle implantation gives us a novel framework for toughening brittle oxide glass.

## Methods

### Glass sample preparation and observation

Soda-lime silica glass with chemical composition of 72SiO_2_-1Al_2_O_3_-6MgO-9CaO-12Na_2_O shown by molar percent was used in this study. The base glass is commercially available product of AGC Inc. In this paper, we call this glass, SLG. For implantation of metal particles, we mixed 3 wt% copper flake into tin bath. The “electrofloat method” was done as the following way: The SLG sample with thickness of 7 mm was put onto a Boron Nitride holder whose bottom side was wide open. A carbon plate was put onto the upper side of the glass to be used as a negative electrode. The sample holder and the tin bath were inserted into a chamber where the atmospheric gas was 5% H_2_ and 95% N_2_. At first, the sample holder and the tin bath was isolated from each other, at room temperature. The tin bath was pre-heated up to 1273 K. Then, the glass was gently approached onto the molten tin bath. As a negative electrode, a carbon plate was placed onto the upper side of the sample glass. A positive electrode stick was inserted into the tin bath and DC-voltage of 2.0 V was applied between the electrodes for 300 sec. Changing the experimental conditions, such as temperature of the tin bath, applied voltage, voltage-holding time changed the density and the size of the Cu particles, and the depth of the particles and Cu ions [To be published]. The reference SLG glass was prepared under exactly the same condition without applying voltage. Therefore, the reference sample had no Cu metal particles nor ions inside the glass. Cooling was done by switching off the electric heater. The cooling rate was around 180 K/min from 1273 K to 873 K. Under 873 K, the holder was isolated from the tin bath and cooled in the atmosphere in air. Before the following measurement, the surface of the glass sample where carbon plate had been attached was polished off, in order to obtain the flat surface which is parallel to the metal-implanted surface. The metal implanted surface was saved from attaching by covering it with a film. After finishing the polishing of the carbon side of SLG, the film was taken off and cleaned with ethanol.

For the measurement of Vickers hardness, *H*_v_, and the crack initiation load, CIL^31^, the surface which attached the tin bath was tested. The sample was polished into the thickness of 3.0 mm perpendicular to the tested surface. *H*_v_ and CIL studies are done by using Vickers diamond indenter. Glass samples were indented in air, (302 K, 30% relative humidity) for a loading time of 15 sec. After the indenter was removed, the number of the radial cracks appeared at the corners of the indentation footprints were counted using optical microscope. Only radial cracks are counted for determining the crack generation, because the cracks normal to glass surface are critical to the total fracture of glass. The percentage of crack initiation was obtained by dividing the number of the corners with the generated cracks by the total number of the corners of indentations. The applied load was increased step-by-step and at least ten indentations were made for each applied load. The hardness was determined with Vickers indentation load of 100 gf for each samples. This load was determined so as to avoid cracking for all the studied glasses^19^. For cross-sectional crack extension observation test, the thickness of the transmission direction of the sample was polished down to 2.0 mm. With this thickness, the sample was able to stand alone while the indenter is inserted into the glass surface. The *in-situ* observation of crack propagation is done by using horizontally aligned light, polarizer, and the high speed camera, Photron, Fastcam SA4 with scan speed of 0.001 sec while stress is applied. Indentation was done horizontally with load controlled by indentation instrument, Instron (Model:FLC-ARS9000 by Future-Tech Corp. Japan).

### Molecular dynamics simulations

To model the composite material composed of copper particles and the soda-lime silica glass, reactive force field (ReaxFF)^23^ was employed because it allows us to calculate interatomic interaction between the heterogeneous materials. Since parameter set for the multicomponent glass experimentally studied is not available in the ReaxFF, we presumed that the composite models are composed of one or several spherical copper particles and amorphous silica (*α*-SiO_2_) matrix instead of SLG. The parameter set, which has been applied to study hydration of Cu-SSZ-13 Zeolite by Psofogiannakis *et al*.^24^, was utilized to simulate the metal-glass boundaries. To examine the volume ratio effect on the mechanical properties, we compare 3, 4 and 5 nm size of nanoparticles. The volume ratio of the copper particles are about 5, 11 and 22% for the models whose particle diameter was 3, 4 and 5 nm, respectively. In addition, multiple particles embedded models, which have apploxinately the same volume ratio with 5 nm particle model, including four 3 nm particles or two 4 nm particles were investigated to unravel effect of particle size on the mechanical properties.

The procedure to obtain the composite model is as follows: Firstly, *α*-SiO_2_ model was constructed by conventional classical MD method using the Teter potential^32^. The initial configuration composed of 19,773 atoms was melted at 3500 K for 500 ps, then it was cooled down to room temperature with cooling rate of 1 K/ps. At temperature more than 2000 K, the canonical ensemble (NVT) was adopted, whereas the isothermal-isobaric ensemble (NPT) was employed at temperature less than 2000 K. Secondly, the interatomic potential was switched from the Teter potential to the ReaxFF potential. Following to the relaxation simulation at 1273 K for 700 ps, the system was cooled down to 300 K with cooling rate of 4 K/ps and then equilibrated at room temperature for 200 ps. The side length of the *α*-SiO_2_ model is about 6.7 nm. After that, as in the case of our recent study^12^, one or several hollows whose diameter are slightly larger than the metal particle were created, and then spherical crystalline copper particles were embedded into the matrix glass model. The combined models were fully relaxed at 1773 K for 1.5 ns, then they were cooled down to 300 K. The cooling rate was 4 K/ps for all cases. NPT ensemble was employed for the all simulations with ReaxFF.

All MD simulations were performed by using LAMMPS package (Large-scale Atomic/Molecular Massively Parallel Simulator)^33^. Time steps for ReaxFF model and Teter model were 0.25 fs and 1.0 fs, respectively. Nosé-Hoover thermostat^34^ and barostat proposed by Tuckerman *et al*.^35^ were employed to control temperature and pressure, respectively.

### Peridynamics simulations

We utilized ordinary state-based Peridynamics^36–39^ to examine the role of the inclusion of copper nanoparticles in the SLG to the fracture resistance via Hertz indentation from the macroscopic point of view. The Peridynamics model for the composite consists of two layers with different elastic properties. The bottom layer with 1 mm thickness represents the SLG, and the top layer of 100 μm thickness stands for the SLG with nanoparticles dispersed. For the Peridynamic framework continuum approximation is imposed. As a result, those layers have lost any atomic information. In particular, since the top layer is homogenized, the nanoparticles and glass matrix are viewed as a single homogeneous material. For implementation of Peridynaimcs macroscopic material properties are required. The properties of elasticity and brittle fracture for each homogeneous material are described by Young’s modulus *E*, Poisson ratio *ν*, and fracture toughness *K*_IC_. To evaluate the improvement of the toughness of the composite arising from the presence of the nanoparticles, plasticity is not taken into account for the sake of simplicity. This assumption is a reasonable condition since Hertz indentation tests induce cracks in the SLG due to the fact that the large contact area formed between the blunt indenter tip, and the glass surface tends to fracture without plastic flow.

The boundary conditions imposed on the indented glass and composite are as follows: the displacement of the bottom surface are constrained in all directions, whereas the side walls are free to move in any direction. A rigid spherical indenter was intended to the glass surface with a constant displacement of 5 m/s. The contact between the indenter and the glass plate was modeled by short range force with a sprint constant of 1 × 10^10^ N/m. The glass model is composed of 570,000 material points in total, resulting from a uniform discretization spacing Δ*x* = 20 μm in all directions. The horizon within which interactions between material points are computed is set at 61 μm, which is slightly longer than 3Δ*x*. Peridynamics simulations were conducted using Peridigm^40^ and FRAXST package^41^ was used for modeling. Discrete momentum balance equations are solved with an explicit numerical scheme with a time step of 3 × 10^−9^ sec, which satisfies the Courant stability condition. Simulation results were visualized by Paraview.

## Supplementary information

Supplementary information
