# Supplementary material for: Higher Toughness of Metal-nanoparticle-implanted Sodalime Silicate Glass with Increased Ductility
Source: Sci Rep. 2019 Oct 28;9:15387. doi: 10.1038/s41598-019-51733-5 (PMC6817821; doi:10.1038/s41598-019-51733-5)

Supporting Information

**Higher Toughness of Metal-nanoparticle-implanted Sodalime Silicate Glass with increased ductility**

**Madoka Ono**^1,2*^**, Satoshi Miyasaka**^3^**, Yoichi Takato**^3^**, Shingo Urata**^3^**, Haruhiko Yoshino**^3^**,**

**Ryota Ando**^3^**, and Yasuo Hayashi**^3^

^1^ AGC Inc., Materials Integration Laboratories, Yokohama, 221-8755, Japan

^2^ Hokkaido University, Research Institute for Electronic Science Laboratory of Nanostructured Functional Materials, Kita 20 Nishi 10, Kita-ku, Sapporo 001-0020, Hokkaido, Japan

^3^ AGC Inc., Innovative Technology Laboratories, Yokohama, 221-8755, Japan

[*madoka-ono@agc.com](mailto:*madoka-ono@agc.com)

Figure S1. Atomic counts measured by EPMA measurement.

Figure S2.

Young’s modulus for various glass with different compressive stress induced by ion-exchange treatment. The Young’s modulus is measured by picodenter. The right figures show load-displacement curves change due to ion-exchange and the difference in the values of their compressive stress.

Figure S3.

Examples of Vickers indentation footprints onto Cu-nano-particle implanted SLG compared to reference SLG.

Figure S4.


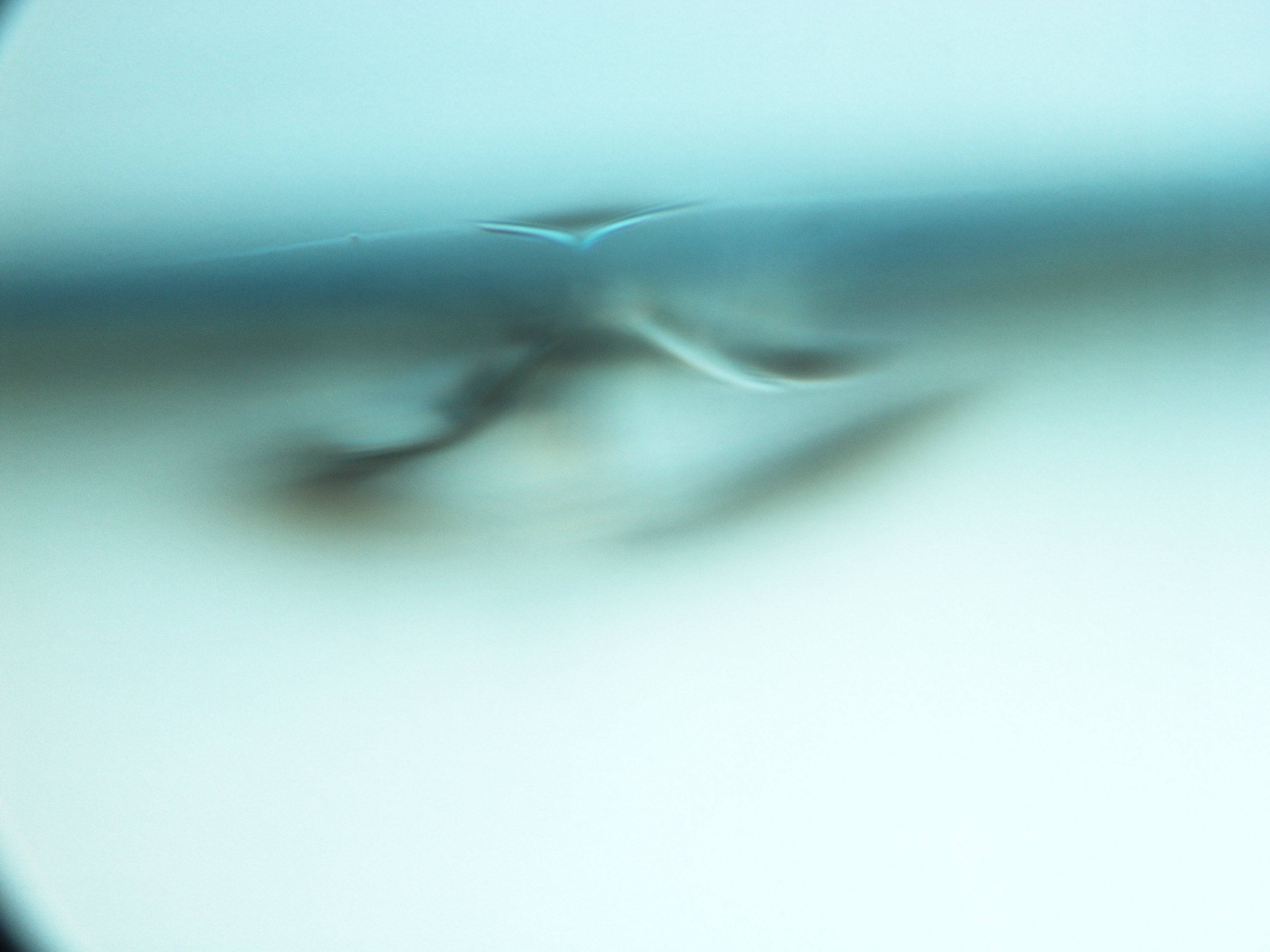

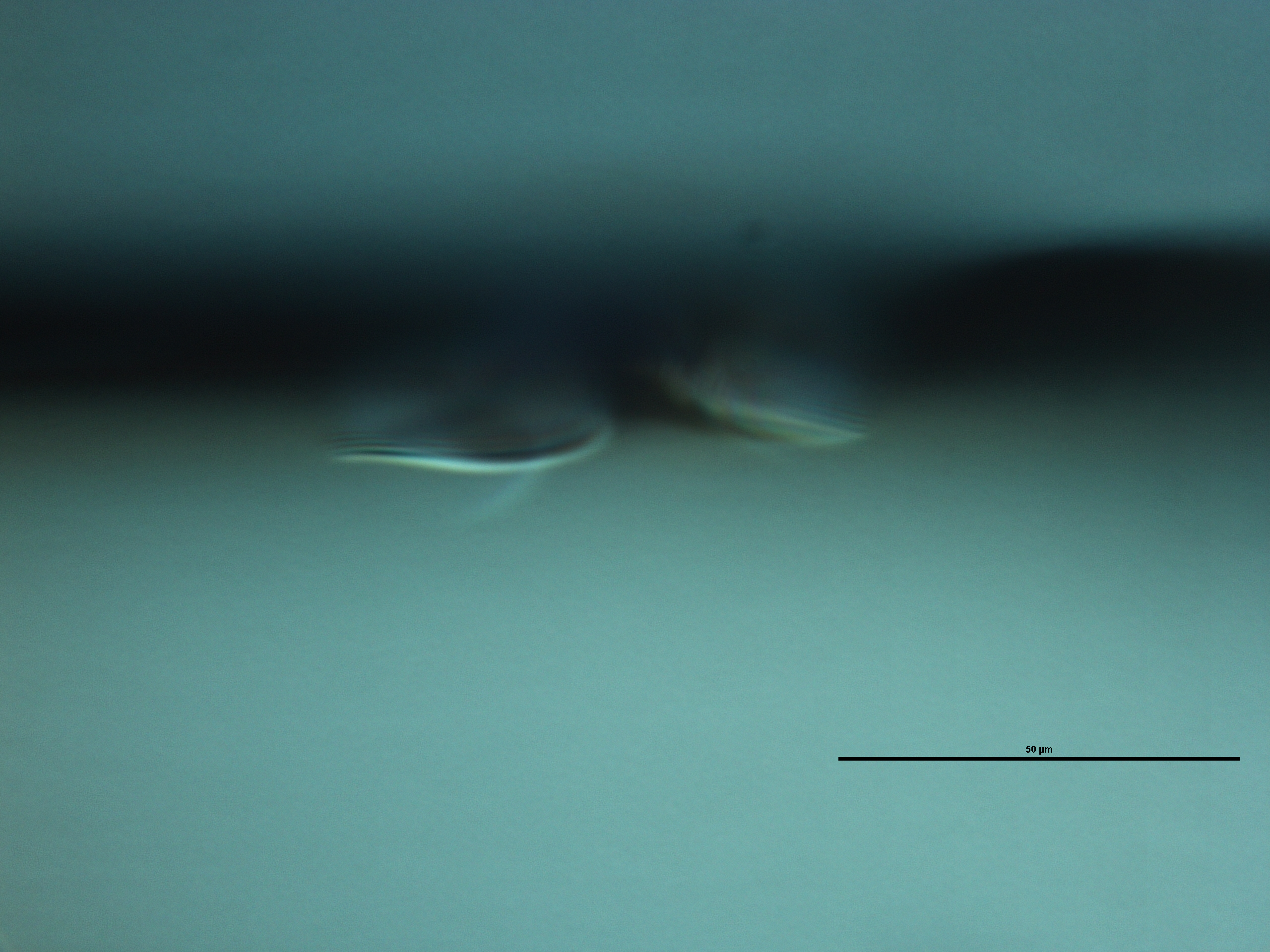

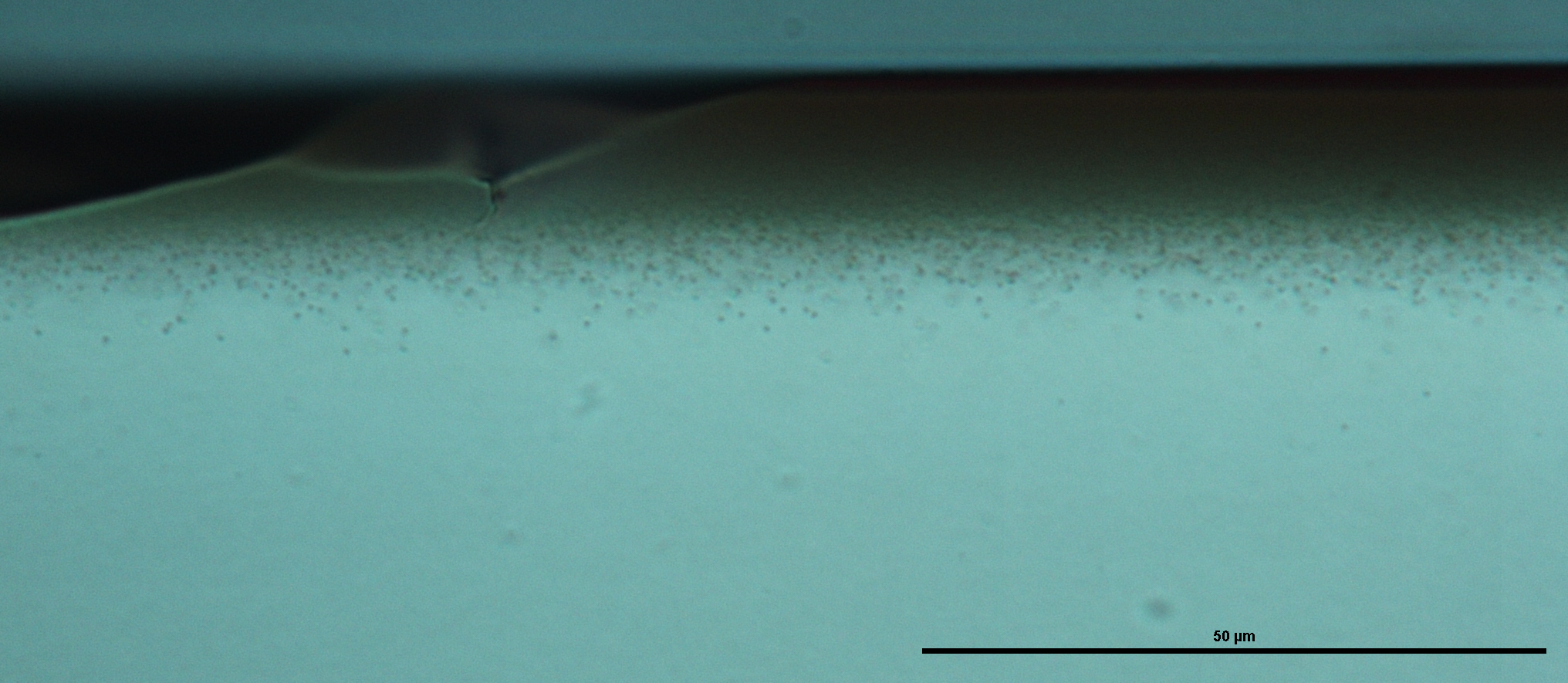
Load 1.0 kgf Load 0.7 kgf Load 0.5 kgf

Examples of the crack propagation observed from cross-sectional view by optical microscope when metal-nano-particle implanted SLG is loaded 1.0, 0.7, and 0.5 kgf, respectively. It is very hard to observe the crack propagation clearly because the crack extends three dimensionally and the focus is onto two dimensional plane. However, it is clearly seen that the cracks are categorized as median crack which is similar as reference SLG. As are obviously seen, the cracks stop when it reaches the metal implanted layer, indicating crack propagation is interrupted by the metal nano-particle implanted layer. It is the effect of the metal-nanoparticle which stops the crack propagation.

Figure S5.

To unravel the deformation mechanism of the nano-scale particle implanted models, we have measured local deformation in the atomistic model at each copper atom position under shear deformation. The figure above displays local deformation gradient, **F**_12_, for three models with 3, 4 and 5 nm diameter models when shear strain is 0.25. We can find that the 4 and 5 nm models clearly demonstrate shear band in the middle of the nano-scale copper particle, indicating that even the models with nanometer-scale particles are large enough to consider dislocation in the metal particle. Thereby, we can infer that the larger metal particles observed in our study should have more profound effect to soften the hybrid glass. Note that the spherical particles in the above figure are copper atoms, and the color shows local deformation gradient at each copper atom when shear deformation **F**_12_ is 0.25.


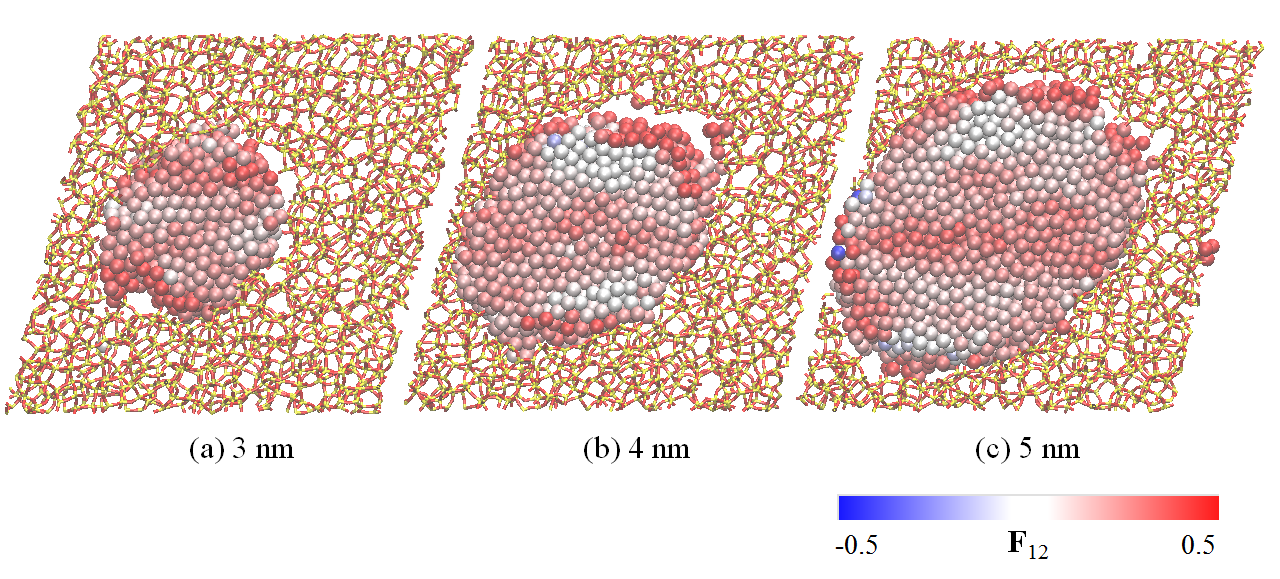

Supplement: Supplementary file 1 — Supplementary information [file 41598_2019_51733_MOESM1_ESM.docx]
